# The Book World of Medicine and Science

**Published:** 1897-07-17

**Authors:** 


### The Book World of Medicine and Science.

Autoscopy of the Larynx and the Trachea. (Direct Examination Without Mirror.) By Alfred Kirstein, M.D., Berlin. Authorised Translation by Max Thorner, A.M., M.D., Cincinnati. With twelve illustrations. One volume, crown octavo, pages xi, 68. Extra cloth. (The F. A. Davis Co., publishers, Philadelphia; New York; and Chicago.)

When Kirstein recently introduced his method of direct inspection of the larynx to the notice of laryngologists his favourable advocacy of the autoscopic method met with healthy scepticism in many quarters. But in all that he claimed he has been corroborated by many observers who have utilised his instrument. This small volume, therefore, will be welcomed as a simple, short, and temperate account of the method and scope of autoscopy, and we cannot doubt that ere long it will be as freely employed by practitioners as laryngoscopy. The translator is to be congratulated on his good work in rendering the author's brochure available to English-speaking medical men.

Clinical Manual of Mental Diseases. By A. Campbell Clark, M.D., F.F.P.S.G. University series. (London: Baillière, Tindall, and Cox. 1897. Price 10s. 6d.)

This is written as a book for practitioners and students, and is essentially clinical throughout. Of pathological teaching there is none, for, as the author says in his preface, the pathology of insanity has yet to be written, and, in fact, the mind is dealt with throughout the book as an entity in itself, free from all the trammels both of physics and metaphysics.

To commence with, the general statements are made that the foundation of the mental constitution is sensation, that sensation and voluntary motion connect mind with its environment, and that in this way man receives impressions from without and responds to them. We are then told that the mental constitution has four parts that are quite familiar: (1) The intellect, (2) emotion, (3) the moral nature, (4) the will and impulses. Of course this is elementary, and to speak of will as a faculty by itself, presiding over the other mental faculties, savours somewhat of the old-fashioned. Yet it is appropriate enough that a book which puts on one side all questions as to the physical conditions of the brain involved in brain diseases should also decline to dissect too finely the elementary stimuli, of which will is the resultant. Moreover, as the author says, it is convenient in clinical practice to think thus of will as presiding over the other faculties, guiding their exercise, determining conduct, and regulating impulses. At any rate, this impersonation of the faculties is a simple way of feeling that we know all about them. Very interesting chapters are given upon sleep, on the diagnostic character of insanity, and on its causation, prognosis, and treatment, after which come a series of chapters dealing separately with the different varieties of mental disease, and finally a very useful one on its legal and civil aspects, including copies of the various forms of certificate which may have to be filled up by medical men and others.

All these chapters are very well done, the descriptions are good and eminently readable, and they are followed in each case by clinical illustrations of the maladies described. It is impossible, however, to get a correct impression of so wide a subject as insanity by viewing it from one side alone. The author, then, has done well not to confine himself to a description of the various forms taken by mental disease, but to describe also the different groupings of mental states which are more especially apt to occur in conjunction with various abnormal bodily conditions. Thus, as well as chapters on mania, melancholia, anergic stupor, dementia, moral insanity, and general paralysis, which may be taken as representing some of the different forms, we have others also on alcoholic insanity, syphilitic insanity, insanity of pregnancy and of the puerperal period, of lactation, of gout, of Bright's disease, of asthma, of cardiac disease, of influenza, and on insanity concomitant with many other conditions—different types of insanity met with in daily practice. Altogether we have read the book with much pleasure, and we feel sure that the student or practitioner who will carefully study its pages will obtain a very useful introduction to the clinical side of the great subject with which it deals. A word of praise should also be said of the book from the printer's and the binder's point of view, for it is a most presentable volume.

Excretory Irritation. By David Walsh, M.D., Edin., Physician Western Skin Hospital, &c. (London, 1897. Baillière, Tindall, and Cox. Pp. 68. Price 3s. 6d.)

The Continental custom of printing graduation theses is happily rare in this country, and the book under notice cannot but add to our gratification that the leniency of British examiners so seldom recoils upon the heads of their fellow practitioners. The author classifies the determining causes of skin diseases under the heading of (1) external irritants, (2) internal irritants, and (3) other internal determinants—whatever that may mean. The internal irritants, to which the essay is devoted, may be drugs, normal excretory products accumulated in the blood, or specific disease poisons. The accumulation of abnormal excretory products as in uræmia, acetonæmia, and cholæmia is entirely unnoticed in this classification, though the first-named condition is referred to a little later. Dr. Walsh appears to believe that a large number of rashes are produced by the direct excretions of various substances through special regions of the skin. No experimental or clinical evidence is given. The only pathogenic micro-organisms which he discusses are those of scarlet fever and measles, neither of which is well within the common ken; his description of the former flourishing in the blood suggests a special revelation. The description of the scratching of a scabies into an eczema is picturesque but hardly pathological, and we fear that accuracy has occasionally been sacrificed to effect in the spelling of foreign proper names. Of the other errors in the book it is only charitable to suppose that a considerable number are misprints.

